# Sustainability in the Development of Natural Pigment-Based Colour Masterbatches and Their Application in Biopolymers

**DOI:** 10.3390/polym16152116

**Published:** 2024-07-25

**Authors:** Ana Ibáñez-García, Raquel Berbegal-Pina, Rosario Vidal, Asunción Martínez-García

**Affiliations:** 1 AIJU Technological Institute for Children’s Products & Leisure, 03440 Ibi, Spain; raquelberbegal@aiju.es (R.B.-P.); sunymartinez@aiju.es (A.M.-G.); 2 Department of Mechanical Engineering and Construction, Green Investigation and Development, Universitat Jaume I, Av. Sos Baynat s/n, 12071 Castelló, Spain; vidal@uji.es

**Keywords:** natural pigment, biodegradable, colour masterbatch, additive, spirulina, curcumin, beetroot, chlorophyllin

## Abstract

This article is focused on the development and characterization of a series of biodegradable and eco-friendly colour masterbatches (MBs), based on natural pigments and biodegradable polylactic acid (PLA) and polybutylene succinate (PBS). Four commercial natural pigments were used, spirulina, curcumin, beetroot and chlorophyllin, to develop the colour masterbatches using a twin-screw extruder. The natural pigment-based MBs were added at 2, 4 and 6 wt%, as additives to study the effect on the properties of injected biodegradable parts (PLA and PBS). The injected samples were characterized in terms of their mechanical (tensile and Charpy impact tests) and visual properties (according to CieLab). In addition, the ageing of the coloured material was followed by colorimetric analysis after its exposure under a Xenon lamp. The mechanical results showed that the addition of coloured masterbatches in different percentages (2–6 wt%) did not significantly change the properties of the materials with respect to the as-received ones. A noticeable colour difference in the injected samples was observed after the first 50 h of artificial light exposure. Regarding environmental concerns, the study showed that the carbon footprint of natural pigments and electricity consumption during extrusion and pelletizing were lower.

## 1. Introduction

Growing concern about the environmental impact of plastic materials has provoked research and development into more sustainable alternatives in a wide variety of sectors, such as packaging, agriculture, consumer goods, etc. In this context, the use of biopolymers, which are plastics derived from renewable resources or that can biodegrade into natural substances such as water, carbon dioxide, and compost, has gained considerable attention.

The ability to produce bioplastics worldwide is expected to rise dramatically, from 2.18 million tonnes in 2023 to 7.43 million tonnes in 2028 [1]. However, to expand their applications and uses, bioplastic formulations require a variety of functional and structural additives.

The use of natural additives is a growing trend in the plastic industry, especially in biopolymers. Several investigations have been carried out to study the addition of natural compounds looking for a specific functionality. For example, naturals fibres such as hem, flax, kenaf, jute and almond shell are used to improve the mechanical strength and stiffness of different biomaterial matrixes, as well as offering a wood-like appearance [2,3,4,5,6,7]. Several studies have developed biocomposites based on biodegradable thermoplastic matrixes and natural fibres in which different vegetable oils have been added to improve the processability, the mechanical ductile properties of biopolymer/lignocellulosic materials and the matrix/fibre compatibility [8,9,10,11,12,13]. Citric acid, glycerol and natural oils have been used as natural plasticizers to increase the flexibility and improve the mechanical ductile properties of biopolymers such as poly(lactic acid), poly(3hydroxybutyrate) and starch thermoplastic [14,15,16,17,18,19,20,21]. Additionally, this additive improves lubrication during the manufacturing process. Plant extracts such as rosemary and green tea, rich in phenolic compounds, are used as natural antioxidants to prevent the oxidative degradation of polymers [22]. For food packaging applications, additives such as essential oils (e.g., oregano oil, clove oil) are used as they possess antimicrobial properties [23].

Natural pigments, obtained from different sources like animals, plants and agricultural waste, are used as colourants mainly in the textile, food and cosmetic industries [22,24,25,26,27]. They offer an environmentally friendly and healthy alternative to synthetic dyes in polymer colouring, with additional benefits that go beyond their function as dyes. In addition, they are biodegradable and are produced from renewable sources.

Carotenoids, such as astaxanthin and beta-carotene, are found in many fruits and vegetables. They are known for their vibrant colours, ranging from yellow to red. These pigments are used in polymers because of their antioxidant properties and their ability to provide intense and stable colours [28]. Anthocyanins are water-soluble pigments found in a wide variety of fruits and flowers, such as grapes, blackberries and roses. They provide colours ranging from red to blue, depending on the pH. In polymers, anthocyanins are used for their ability to impart vibrant colours and as well as for their antioxidant benefits [29]. Chlorophyll, the green pigment found in plants, is used to naturally colour polymers. In addition to its characteristic green colour, chlorophyll has antimicrobial properties, making it useful in food packaging applications [29]. Polyphenols, present in tea, cocoa and many fruits, are known for their antioxidant properties. These compounds can also be used as natural colourants in polymers, providing colours ranging from yellow to brown. Some microorganisms produce natural pigments that can be used in the colouring of polymers. For example, bacterioruberin, produced by halophilic bacteria, provides an intense red colour and has antioxidant and antimicrobial properties [28].

Currently, the packaging industry is focusing on the application of natural pigments in polymers to develop smart packaging with antioxidant and antimicrobial properties. Researchers have focused on developing smart packaging material based on natural pH-sensitive pigments immobilized in biopolymers for food freshness monitoring in real time [23].

The incorporation of natural pigments into biodegradable matrices for the production of injection moulded parts represents a promising innovation in the plastics industry. This technique not only has the potential to reduce the environmental impact of plastic production but can also improve the safety and sustainability of the final products. However, it also presents challenges that must be addressed through continuous research and development to optimize its application and commercial viability. Pigments derived from natural sources, such as plants and minerals, are less toxic and have a lower environmental impact than synthetic pigments. Incorporating natural pigments may require adjustments to the injection moulding process, which may lead to the development of new techniques and technologies in the manufacturing of biodegradable plastics. Furthermore, the study and optimization of the behaviour of these pigments in biodegradable matrices can lead to improvements in the efficiency of manufacturing processes and the quality of the final products.

This article analyzes the processing of natural pigments to obtain natural pigment-based MBs in two biodegradable polymeric matrixes, polylactic acid (PLA) and polybutylene succinate (PBS), and the study of the colouring capacity by the addition, in different percentages, of natural pigments in the polymeric matrix.

## 2. Materials and Methods

### 2.1. Materials

Two commercially available biopolymeric matrices were used to develop the masterbatches, polylactic acid (PLA), Beograde INJ038, supplied by Beologic, (Sint-Denijs, Belgium) and polybutylene succinate (PBS), BioPBSFZ71PM, supplied by Biochem Company (Chatuchak, Thailand). These grades were selected due to being biodegradable polymers in industrial compost conditions and having a relatively low melting temperature, which allows the incorporation of natural pigments, minimizing the risk of their degradation. Table 1 shows some properties of the as-received materials.

Different commercial natural pigments were selected. Table 2 shows the reference and some properties of each of them. All of them were supplied by Coralim.

In addition, titanium dioxide, stearic acid and calcium carbonate were used as ultraviolet (UV) absorber, stabilizer, dispersing agent and lubricant. These components were supplied by Sigma-Aldrich (Madrid, Spain).

### 2.2. Experimental Procedure

#### 2.2.1. Thermal Analysis

The main thermal degradation parameters of natural pigments and natural pigment-based MBs, degradation initial temperature (T_onset_) and maximum mass loss rate temperature (T_max_) were studied by TGA using a TA Instrument Q500 (TA Instruments, New Castle, DE, USA) thermogravimetric analyser. Then, those samples with an average weight between 8 and 10 mg were placed in standard platinum crucibles of 70 µL. In this case, all samples were subjected to the following temperature programme: from 30 to 600 °C under nitrogen (N_2_) atmosphere at a rate of 10 °C/min and from 600 to 1000 °C under oxygen (O_2_) atmosphere at a rate of 10 °C/min with a purge gas flow of 10 mL/min.

#### 2.2.2. Preparation of Natural Pigment-Based MBs

Prior to processing, PLA was dried for 3 h at 60 °C in a Arid X10X dryer (Dri-air Industries, East Windsor, CT, USA) to eliminate its moisture and to avoid hydrolytic reactions [30].

Different formulations of natural pigment-based MBs with each biodegradable matrix and natural pigment were developed by using a TEACH LINE COMPOUNDER ZK co-rotating twin-screw extruder (25:24 L/D) with two gravimetric feeders (COLLIN Lab & Pilot Solutions GmbH, Maitenbeth, Germany) a main one for the polymeric material and a secondary one for fillers, additives and pigments in powder form. Before feeding, manual pre-mixing of the different components, 30 wt% titanium dioxide (TiO_2_), 10 wt% stearic acid, 40 wt% natural pigment and 20 wt% calcium carbonate (CaCO_3_), was carried out and fed at 20 wt% into the extruder thorough the secondary feeder. The temperature profile (from feeding zone to die) was set as follows: 50-140-145-145-145-145 °C for masterbatches based on PBS and 50-180-185-190-190-190 °C for masterbatches based on PLA. The rotating speed was 65 rpm. The feed rate into the extruder was 2 kg/h.

#### 2.2.3. Melt Flow Rate (MFR)

The determination of the melt flow rate (MFR) was carried out according to the ISO 1133 standard [31] with MFI TWEL VEINDEX equipment (ZwickRoell, Barcelona, Spain) at 190 °C and 2.16 kg. The cut time between two consecutive measurements was 15 s.

#### 2.2.4. Injection Moulding

PLA and PBS dogbones-standardized samples with different percentages (2, 4, 6 wt%) of the developed natural pigment-based MB were moulded using an injection moulding machine, DEMAG Ergotech, 110–430 h/310 V (Sumitomo Demag Plastics Machinery GmbH, Schwaig, Germany). The injection conditions used to inject PLA and PBS test specimens are listed in Table 3. Specimens were conditioned at a temperature of 23 °C and relative humidity of 50% for at least 16 h before testing.

#### 2.2.5. Mechanical Properties

The mechanical properties of the injected dogbones were determined to study the influence of the natural pigment-based MBs and their content in the material.

Tensile and flexural tests were performed using an Instron 6025 universal testing machine (Instron, Barcelona, Spain) with 5 kN power sensors. The tensile test was performed according to the ISO 527 standard [32], using a crosshead speed of 1 mm/min for Young’s Modulus determination and 5 mm/min for tensile and elongation at break determination. The extensometer used was MTS 634.11F-54 (MTS Systems Corporation, Eden Prairie, MN, USA). Recorded values included ultimate tensile strength (UTS), Young’s modulus and strain at break. A total of 5 specimens from each material were tested using standardized sample 1A (dogbone).

Impact tests were performed with a Resil 5.5 impact testing device CEAST RESIL IMPACTOR (CEAST, Torino, Italy) with a 5-Joule hammer. Then, test samples were cut and tested according to the ISO 179 standard [33]. A total of 10 samples from each material were tested.

#### 2.2.6. Ageing Test

Colour fastness was evaluated by means of artificial light ageing with a Xenon lamp. The equipment used was a Xenotest ATLAS ALPHA+ (Atlas Material Testing Technology, Mt Prospect, IL, USA). The ageing test was performed according to ISO 4892 [34], method A and cycle nº1 (exposure period: 102 min dry and 18 min under water spraying; chamber temperature: 38 ± 3 °C; black standard temperature: 65 ± 3 °C; relative humidity: 50%; irradiance: 60 W/m^2^). The test duration was 50 h.

#### 2.2.7. Colour Measurements

The colour variation of the samples injected with natural pigment-based MBs after the ageing test was evaluated with a CR-200 Chroma Meter (Konica Minolta Sensing Americas, Inc, Ramsey, NJ, USA). Moreover, the colour indexes (L*, a* and b*) were measured according to the following criteria: L* = 0, darkness; L* = 100, lightness; +a* = red, −a* = Green and +b* = yellow, −b* = blue. From these coordinates, it was possible to determine the colour difference associated with this space. The colour variation, $\Delta E_{ab}^{*}$, was obtained by the following Equation (1) and compared with the colour coordinates of the formulation before ageing.

(1) $$\Delta E_{ab}^{*}=\sqrt{{\left(\Delta L^{*}\right)}^{2}+{\left(\Delta a^{*}\right)}^{2}+{\left(\Delta b^{*}\right)}^{2}}$$

The colour change was assessed according to experimentally verified data [35]: unnoticeable, ($\Delta E_{ab}^{*}<1)$; only an experienced observer can notice the difference $\left(1<\Delta E_{ab}^{*}<2\right)$; an unexperienced observer notices the difference $\left(2<\Delta E_{ab}^{*}<3.5\right)$; there is a clear noticeable difference $\left(3.5<\Delta E_{ab}^{*}<5\right)$; and the observer notices two different colours $\left(\Delta E_{ab}^{*}>5\right)$. Colour variation could be indicative of pigment degradation.

#### 2.2.8. Environmental Concerns

The environmental impacts associated with the production of natural pigments from curcumin and spirulina were evaluated using the Life Cycle Assessment (LCA) methodology, conducted in accordance with ISO standards (14040/14044) [36,37] and compared with the inorganic pigment Green 7. The LCA was performed from cradle to gate for 1 kg of pigment. The inventories of the three pigments were modelled from the literature: yellow is obtained from natural curcumin, considering turmeric cultivation [38] and extraction with acetone in microwaves [39]; blue is obtained from natural sun-dried spirulina [40]; and inorganic pigment is obtained from green copper (II) phthalocyanine [41]

The Ecoinvent 3.9 flows were used for this LCA. The selected flows followed the cut-off system model. The method described in the standard EN 15804 +A2 (adapted) V1.00 [42], based on the European method EF 3.1, included in SimaPro 9.5 software (SimaPro, Amersfoort, The Netherlands), was chosen to estimate the potential climate change impact. Compared to the EF method, EN 15804 +A2 differs in the characterization factors of biogenic CO_2_ uptake and emissions, which were set in the standard as equal to “−1” (CO_2_ uptake) and “+1” (CO_2_ release).

## 3. Results

### 3.1. Development of Natural Pigment-Based MBs

The main thermal degradation parameters of natural pigments and as-received biodegradable matrices (PLA and PBS) were determined by TGA to monitor the thermal stability of natural pigments and polymeric matrices and select the processing temperature profile for developing natural pigment-based MBs. Table 4 shows the degradation temperature (T_onset_) and maximum mass loss rate temperature (T_Max_) of the natural pigments, as well as of the as-received polymers. PBS polymer degraded in a single-step process and showed moderate thermal stability with a T_onset_ of 366.50 °C. Regarding the PLA, its degradation was in two steps; it presented a T_onset_ of 325.07 °C, lower than PBS. Natural pigments presented lower thermal stability than as-received polymers. There were some differences between the natural pigments studied. This is explained by the fact that the thermal stability of natural pigments depends on the chemical composition, like in the case of natural fibres.

According to the thermal degradation parameters shown in Table 4 and the melting temperature of polymers (Table 1), the temperature profile selected to obtain the masterbatches and the injected specimens was established above the melting temperature of the polymer and below the degradation temperature of the natural pigments (temperature profile of PBS: 50-140-145-145-145-145 °C. Temperature profile of PLA: 50-180-185-190-190-190 °C)

Before the extrusion compounding process, the moisture content of the natural pigments was determined using a moisture analyser. The moisture content of all natural pigments was less than 0.03 wt%, so no drying process was required before the extrusion process of natural pigment-based MBs.

**Figure 1:**
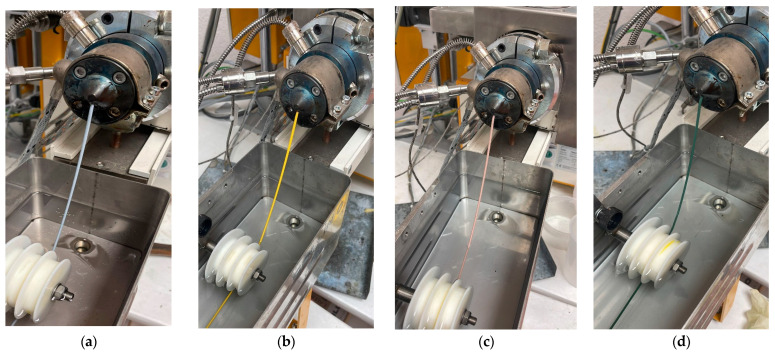
Extrusion process of natural pigment-based MBs based on PBS with (**a**) spirulina, (**b**) curcumin, (**c**) beetroot, (**d**) chlorophyllin.

**Figure 2:**
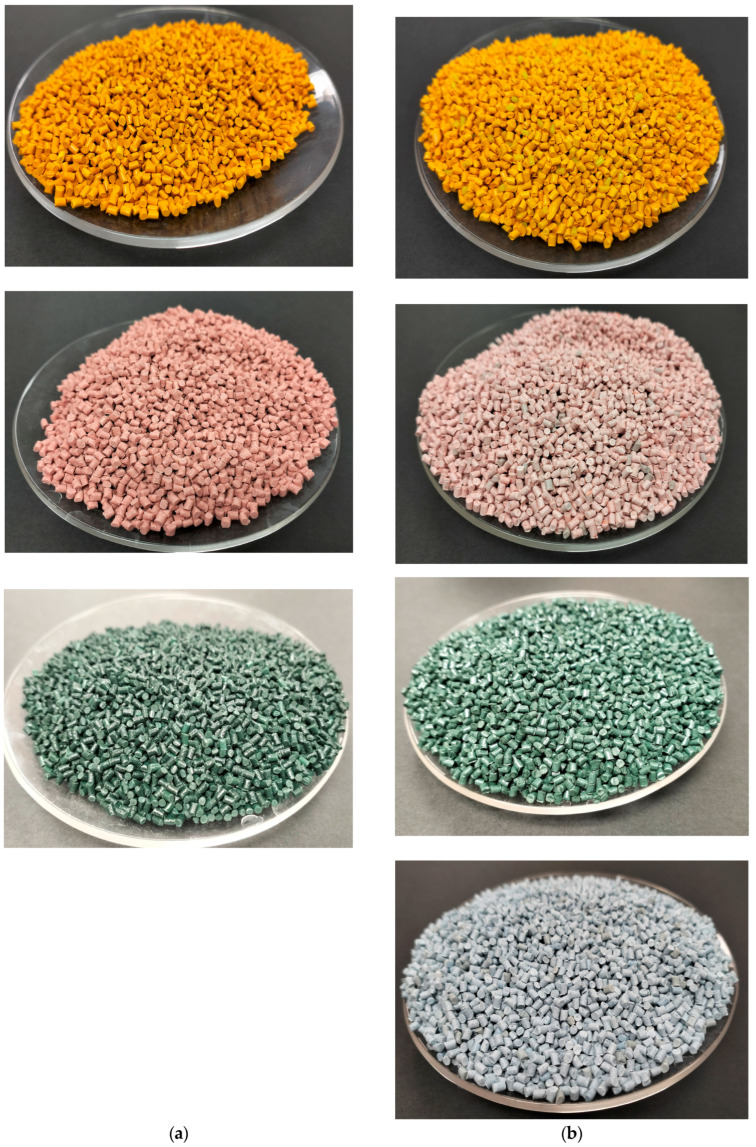
Natural pigment-based MBs developed (**a**) based on PLA matrix, (**b**) based on PBS matrix.

Only in the case of spirulina pigment it was not possible to obtain blue PLA-based masterbatches as the temperature profile during the processing degraded the pigment. The extruded material was finally pelletized using an air-knife. Figure 1 shows the extrusion process and Figure 2 shows the masterbatches obtained.

### 3.2. Characterization of Natural Pigment-Based MBs

**Figure 3:**
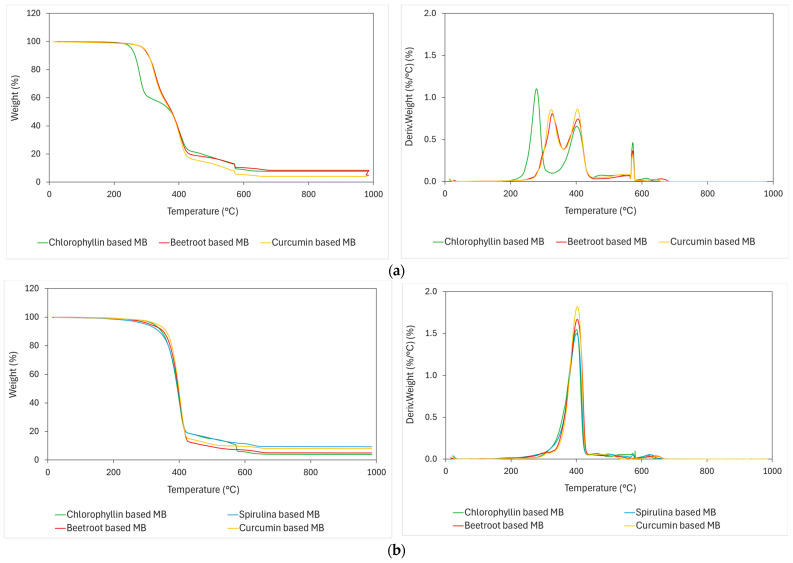
TGA thermograms and first derivative (DTG) curves of natural pigment-based MBs. (**a**) Based on PLA matrix, (**b**) based on PBS matrix.

Figure 3 shows the TGA and DTG curves of natural pigment-based MBs. The addition of natural pigments reduces the thermal stability of the as-received polymers because natural pigments start degradation earlier than the polymer matrix. T_onset_ and T_max_ are moved towards lower temperatures with the addition of natural pigments. As shown in Table 5, T_onset_ changes progressively from 325 and 367 °C for the as-received PLA and PBS polymers, respectively (Table 4), decreasing to values of 258 and 356 °C.

The rheological properties of the natural pigment-based MBs were determined by MFR. Table 6 shows the MFR results obtained from each masterbatch, as well as the as-received polymers. The incorporation of natural pigments slightly decreased the flow rate of the polymer, which meant a slight increase in viscosity.

### 3.3. Characterization of the Injected Specimens with Natural Pigment-Based MBs

**Figure 4:**
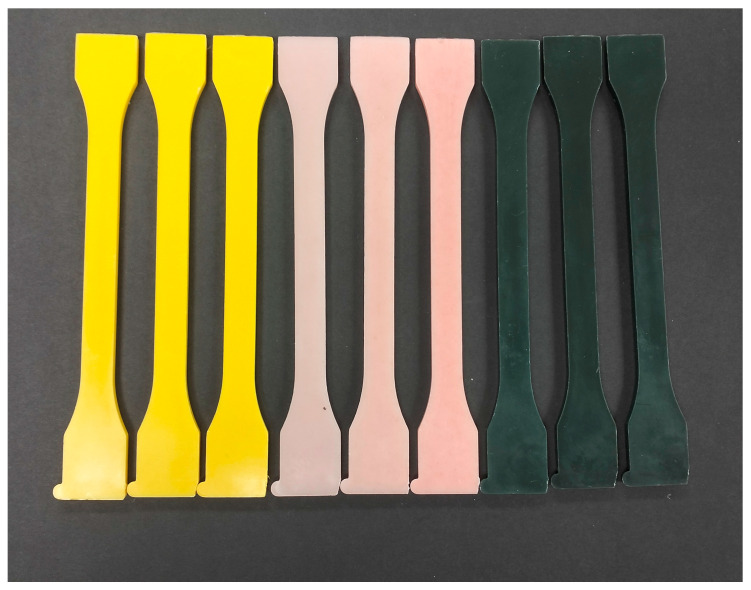
Visual aspect of the injected PLA specimens with 2, 4 and 6 wt% of natural pigment-based MBs developed.

**Figure 5:**
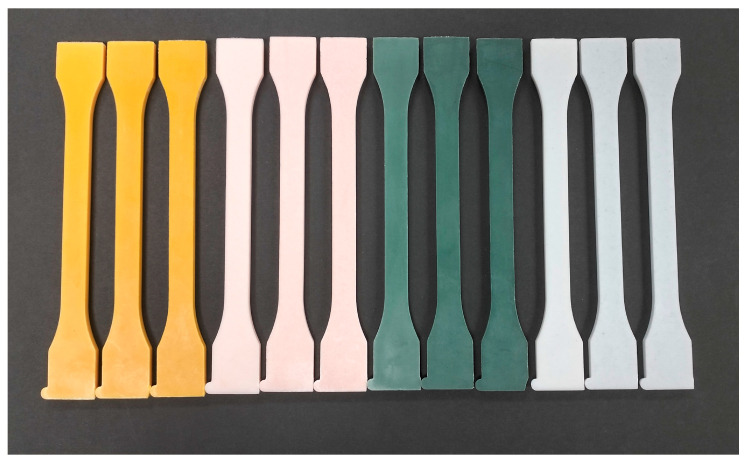
Visual aspect of the injected PBS specimens with 2, 4 and 6 wt% of natural pigment-based MBs developed.

The appearance of the injected specimens of PLA and PBS with the different natural pigment-based MBs added between 2 and 6 wt% are shown in Figure 4 and Figure 5, respectively.

Table 7 summarizes the colour indexes (L*, a* and b*) and the colour variation measured by $\Delta E_{ab}^{*}$ with respect to the samples with the 2 wt% colour masterbatch.

The increment in colour masterbatch concentration in the biopolymer matrix had an effect. As expected, the $L^{*}$, a* and b* coordinates changed progressively. The colour variation clearly showed an increasing tendency. The $\Delta E_{ab}^{*}$ was more remarkable and noticeable for the PBS injected samples.

### 3.4. Mechanical Properties

**Figure 6:**
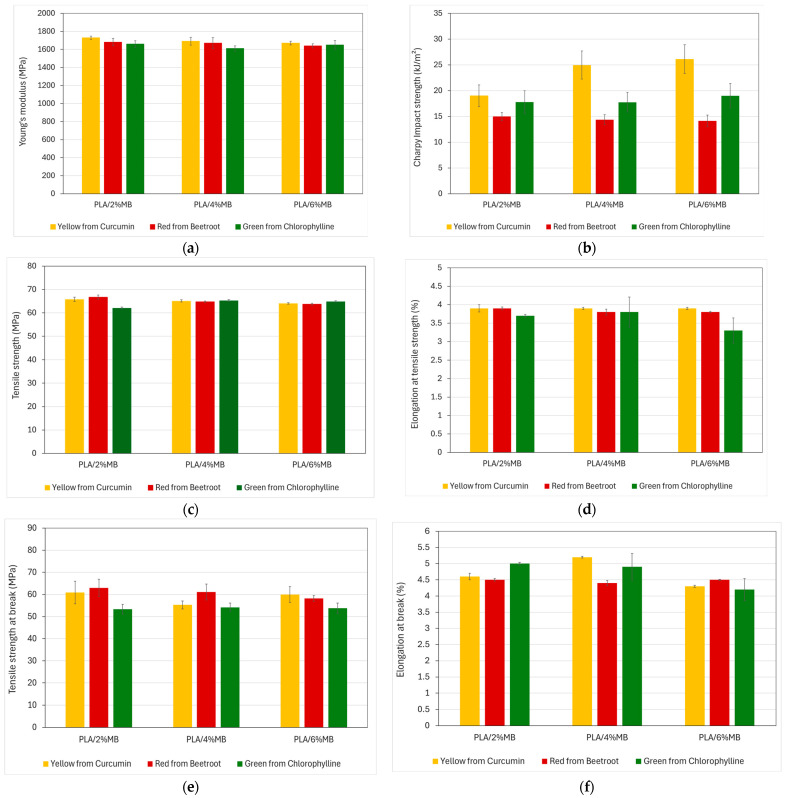
Mechanical properties of injection-moulded samples of PLA with natural pigment-based MBs added in different percentages. (**a**) Young’s modulus, (**b**) Charpy impact strength, (**c**) tensile strength (σ_M_) and (**d**) elongation at tensile strength (Ɛ_M_), (**e**) tensile strength at break (σ_r_), (**f**) elongation at break (Ɛ_R_).

Table 8 shows a summary of the tensile properties and Charpy impact strength of all developed PLAs with different concentrations of colour masterbatches. The as-received PLA presented a Young’s modulus of 1710 MPa and a tensile strength of 65.6 MPa. As can be seen in Table 8, the addition of the developed natural pigment-based MBs did not have an influence on tensile properties. Regarding impact strength, the as-received PLA presented an impact strength of 18.31 kJ/m^2^. This value ranged from 14.98 kJ/m^2^ to 26 kJ/m^2^. The lowest value was found in the samples incorporating the colour masterbatch with beetroot extract. On the other hand, the samples with the highest impact resistance were those incorporating 4–6 wt% of MB with curcumin extract. However, the addition of the natural pigment-based MBs had practically no influence on the final material properties. Similar results were obtained in a study in which 3 wt% of MB based on an organic protective photoluminescent pigment was incorporated into PLA, which also showed that the addition of the pigment did not impact the mechanical properties of PLA [43]. In another investigation, the effect of different conventional pigments, blue 15:1, green 7, pink PR122 and yellow 155, on the properties of dope-dyed PLA multifilament yarns was studied. Pigments and PLA were compounded and added into the PLA matrix in 5 wt%. Mechanical characterization showed that there was no significant change in the mechanical properties of the yarn in the presence of colourants [44]. Figure 6 shows the evolution of tensile properties and impact strength.

**Figure 7:**
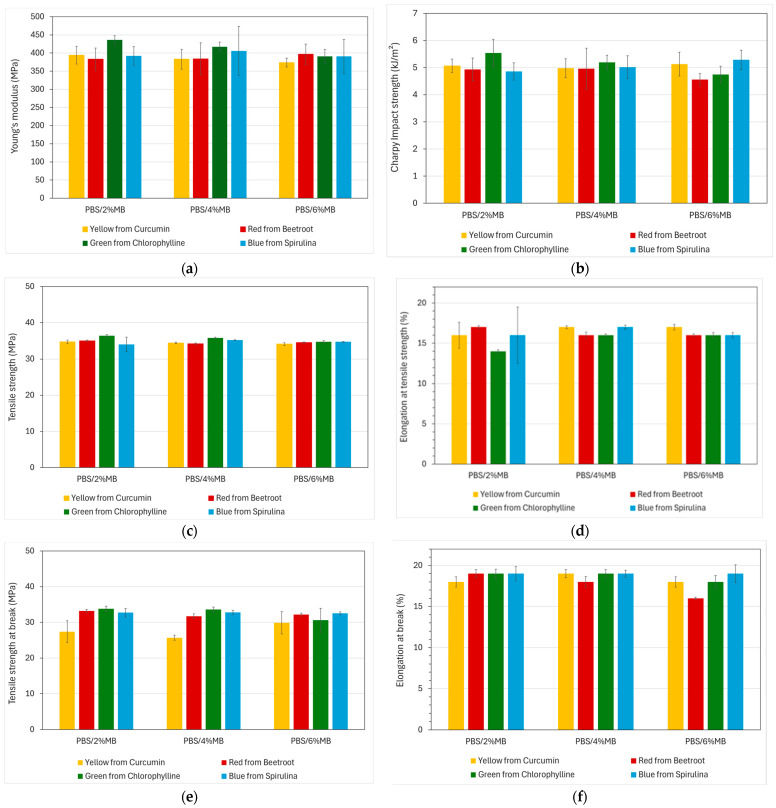
Mechanical properties of injection-moulded samples of PBS with natural pigment-based MBs added in different percentages. (**a**) Young’s modulus, (**b**) Charpy impact strength, (**c**) tensile strength (σ_M_) and (**d**) elongation at tensile strength (Ɛ_M_), (**e**) tensile strength at break (σ_r_), (**f**) elongation at break (Ɛ_R_).

Table 9 shows a summary of the tensile properties and Charpy impact strength of PBS with different concentrations of the developed natural pigment-based MBs. The as-received PBS presented a Young’s modulus of 392 MPa, a tensile strength of 34.5 MPa, and an elongation at tensile strength of 16%. The addition of different percentages of natural pigment-based MBs based on PBS and natural pigments into the PBS matrix showed similar behaviour to PLA samples, with no significant variations in tensile mechanical properties. The PBS without masterbatches of colour presented an impact strength of 5.17 kJ/m^2^. This value varied slightly from 4.75 kJ/m^2^ to 5.54 kJ/m^2^. Analogously to PLA-based samples, the addition of the natural pigment-based MBs had practically no influence on the final material properties. Figure 7 shows the evolution of tensile properties and impact strength.

### 3.5. Ageing Test of Injected Samples

**Figure 8:**
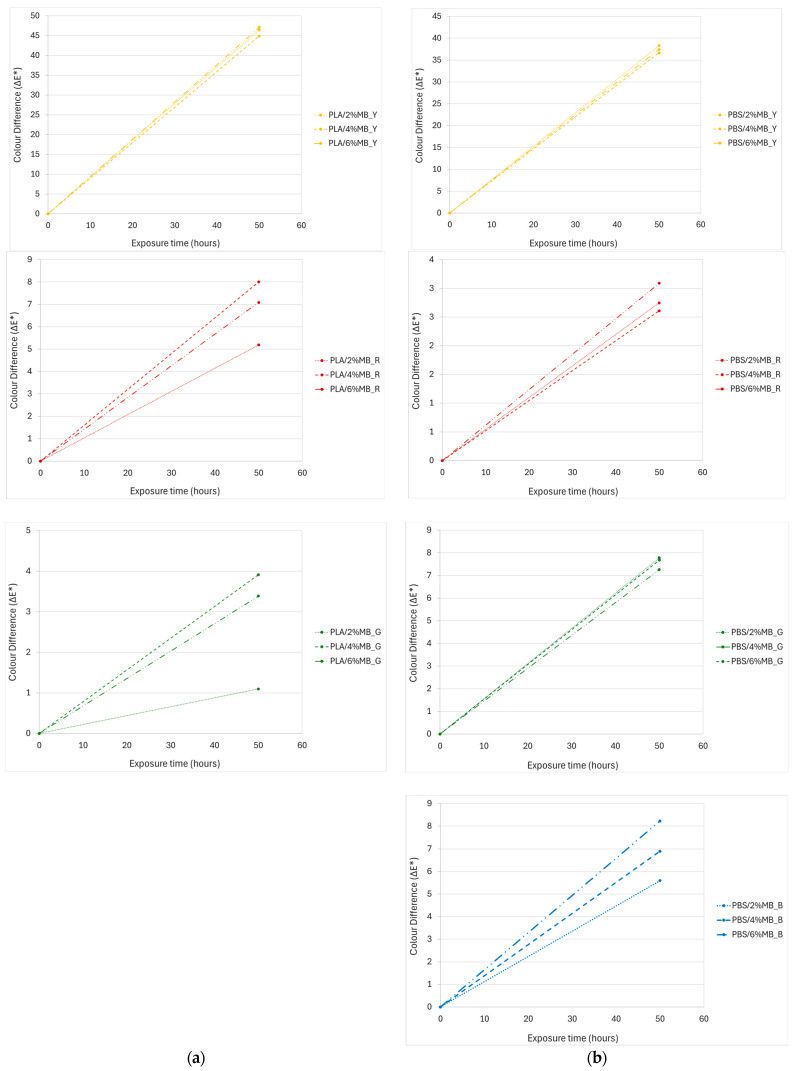
Colour difference in injected samples with different percentages of natural pigment-based MBs. (**a**) PLA injected samples, (**b**) PBS injected samples.

The ageing test of the coloured material was carried out under a Xenon lamp to determine the effect of the UV irradiations on the colouring. Figure 8 shows the change in the colour variation of the injected samples with different content of masterbatches before and after ageing.

The colorimetric measurements (L*, a* and b*) showed a low stability to UV exposure as after only 50 h of testing, the obtained value of $\Delta E^{*}$ was higher than 2, which meant that any unexperienced observer would have noticed the difference. Only in the PLA sample with 2 wt% green masterbatch (PLA/2%MB_G) was the colour difference less than 2. The results obtained are in accordance with those obtained in a previous study where it was determined that natural dyes are sensitive to UV ageing tests and temperature [45].

### 3.6. Environmental Concerns

**Figure 9:**
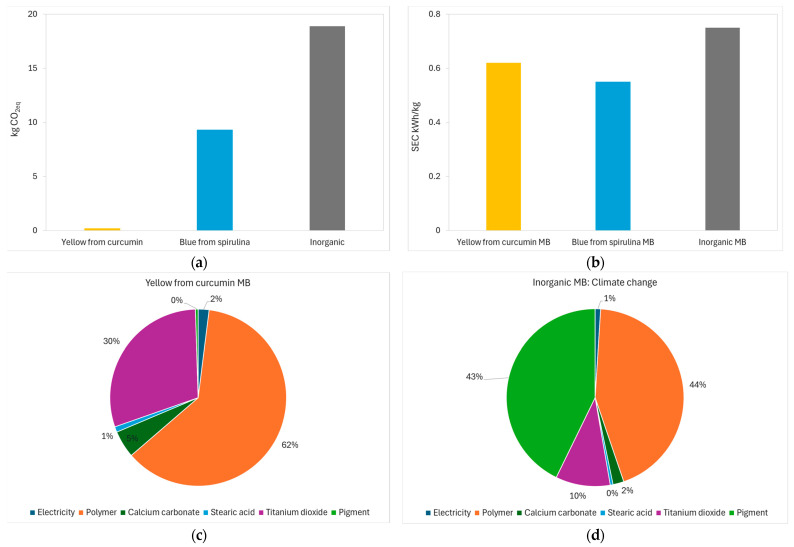
Comparative assessment of pigments and masterbatches. (**a**) Carbon footprint of 1 kg of yellow, blue and inorganic (green) pigments. (**b**) Electricity consumption for the extrusion and palletization of 1 kg of yellow, blue and Inorganic masterbatch. (**c**) Contributions to the carbon footprint of the yellow masterbatch. (**d**) Contributions to the carbon footprint of the inorganic masterbatch.

The reduction in climate change impacts achieved by the two natural pigments with respect to the inorganic pigment is very considerable—see Figure 9a—going from 18.65 kg CO_2_eq in the inorganic pigment to 0.04 kg CO_2_eq in the yellow from curcumin pigment. However, it should be noted that the impacts of natural pigments are very sensitive to the drying processes used, greenhouse gas emissions from energy sources and yields for the CO_2_ sequestration during plant growth.

Electricity consumption for masterbatch extrusion and pelletizing, as described in Section 2.2.1, was measured utilizing the CIRCUTOR AR5 electrical analyser, with a sampling rate of 2 kHz and recording every second. Electricity results are shown in Figure 9b. The inorganic masterbatch was obtained with high-density PE as the polymer and the inorganic green 7 pigment, following the same percentages and the same composition as in the organic masterbatches. The two organic masterbatches exhibited lower power consumption than the inorganic masterbatch, with the blue masterbatch with PBS and spirulina having the lowest power consumption.

The carbon footprint of the masterbatch is strongly influenced by the carbon footprint of the polymer, as this is 80% of its weight, as shown in Figure 9c,d. However, the contribution of the pigment to the carbon footprint can vary from 0% in the case of the yellow masterbatch, as shown in Figure 9c, to 43% in the case of the inorganic masterbatch, as shown in Figure 9d. In both cases, electricity consumption is low, only 0.7% and 1.8%, respectively.

In conclusion, the carbon footprint of natural pigments derived from curcumin and spirulina has been shown to be lower than that of the inorganic green pigment. Electricity consumption during extrusion and pelletizing has also been shown to be lower. Finally, in organic masterbatches, the contribution of the carbon footprint from natural pigments is lower than the contribution of inorganic pigments in inorganic masterbatches.

## 4. Conclusions

This study developed a series of biodegradable and eco-friendly colour masterbatches (MBs), based on natural pigments and biodegradable polylactic acid (PLA) and polybutylene succinate (PBS). Four commercial natural pigments were used, spirulina, curcumin, beetroot and chlorophyllin.

Natural pigments presented lower thermal stability than as-received polymers. There were some differences between the natural pigments studied. This is explained by the fact that the thermal stability of natural pigments depends on the chemical composition.

The processing of the developed natural pigment-based MBs were successfully carried out without problems by using conventional extrusion-compounding and injection moulding equipment at temperatures below the degradation point of the natural pigments and above the melting temperature of the polymer.

The incorporation of natural pigments slightly decreased the flow rate of the polymer, which meant a slight increase in viscosity.

The natural pigment-based MBs were added at 2, 4 and 6 wt%, as additives, to study the effect on the properties of injected biodegradable parts (PLA and PBS). The injected samples were characterized in terms of their mechanical (tensile and Charpy impact tests) and visual properties (according to CieLab). In addition, the ageing of the coloured material was followed by colorimetric analysis after its exposure under a Xenon lamp.

The experimental results revealed that the addition of natural pigment-based MBs in different percentages (2–6 wt%) did not significantly change the mechanical properties of the materials with respect to the as-received ones. The variation in the percentage of natural pigment-based MBs had a substantial influence on colour. Noticeable variation in the colour of the injected samples was observed after the first 50 h of artificial light exposure.

Regarding environmental concerns, the study demonstrated that the carbon footprint of natural pigments and electricity consumption during extrusion and pelletizing were lower. Furthermore, the contribution to the carbon footprint from natural pigment-based MBs was lower than the contribution of inorganic pigments in inorganic masterbatches.

The use of natural pigments based on masterbatches in the plastic industry presents several potential applications, driven by the growing demand for sustainable and environmentally friendly solutions. Some uses could be, for example, in the packaging sector (food or cosmetic) and the agricultural sector. Natural pigments could be used in biodegradable and compostable packaging materials, ensuring safety for food contact and reducing environmental impact and mulch film.

In addition, other interesting sectors are 3D printing and concretely fused filament fabrication (FFF), since one of the polymers used is PLA. These natural pigment based-MBs would allow for the obtainment of more sustainable colour 3D-printed parts.

## Figures and Tables

**Table 1:** Properties of Beograde INJ038 and BioPBSFZ71PM extract from datasheet supplied by suppliers.

Property	Beograde INJ038	BioPBSFZ71PM
Melting temperature (°C)	165	115
Melt flow rate (g/10 min)	17	22
Density (g/cm^3^)	1.20	1.26

**Table 2:** Natural pigments used.

Property		Extract SP-180	Extract 95%P	Extract RBS-100	Extract 95 HPD-P1
Origin		Spirulina	Curcumin	Beetroot	Chlorophyllin
Colour		Blue	Yellow	Red	Green
Particle size (µm)					
	D10	10.3	1.46	8.04	1.6
	D50	24.5	9.3	33.1	5.42
	D90	44.9	35.1	69.3	22.9
Purity (%)		98	90–100	95–100	95
Heavy metals (ppm)		As < 3, Pb < 5, Hg < 1, Cd < 1	As < 3, Pb < 10, Hg < 1, Cd < 1	As < 1, Pb < 1, Hg < 1, Cd < 1	As < 3, Pb < 5, Hg < 1, Cd < 1

**Table 3:** Injection condition of PLA and PBS with natural pigment-based MB dogbones.

Property	Dogbones Based on Beograde INJ038	Dogbones Based on BioPBSFZ71PM
Barrel profile (°C)	190-190-180-170-35	160-160-150-140-35
Mould temperature (°C)	25	25
Injection speed (mm/s)	60	60
Pack pressure (bar)	600	400
Pack time (s)	10	15
Back pressure (bar)	50	50
Cooling time (s)	40	40

**Table 4:** Thermal properties of the natural pigments and as-received polymers.

Samples	T_Onset_ (°C)	T_Max_ (°C)	Residual Weight (%)
Curcumin	244.19	305.62	0
Beetroot	266.57	304.93	1.42
Chlorophyllin	257.66	298.77	0
Spirulina	228.50	303.83	0
PLA INJ038	325.07	343.49 and 408.53	0
BioPBSFZ71PM	366.50	402.84	0

**Table 5:** Thermal properties of the natural pigment-based MBs.

Samples	T_Onset_ (°C)	T_Max_ (°C)	Residual Weight (%)
Curcumin-based MB_PLA_	299.77	323.68 (T_Max1_) and 403.52 (T_Max2_)	4.104
Beetroot-based MB_PLA_	300.43	326.51 (T_Max1_) and 404.95 (T_Max2_)	8.424
Chlorophyllin-based MB_PLA_	258.35	326.51 (T_Max1_) and 404.95 (T_Max2_)	7.749
Curcumin-based MB_PBS_	369.57	403.27	8.038
Beetroot-based MB_PBS_	366.41	402.82	4.999
Chlorophyllin-based MB_PBS_	356.62	400.65	9.339
Spirulina-based MB_PBS_	356.62	400.24	4.028

**Table 6:** MFR of the natural pigment-based MBs.

Samples	MFR at 190 °C/2.16 kg (g/10 min)
As-received PLA	50.00 ± 4.91
Curcumin-based MB_PLA_	49.36 ± 1.62
Beetroot-based MB_PLA_	49.18 ± 10.56
Chlorophyllin-based MB_PLA_	48.24 ± 28.19
As-received PBS	23.47 ± 0.15
Curcumin-based MB_PBS_	22.39 ± 5.02
Beetroot-based MB_PBS_	22.71 ± 3.92
Chlorophyllin-based MB_PBS_	22.27 ± 4.78
Spirulina-based MB_PBS_	22.80 ± 2.56

**Table 7:** Colour parameters ($L^{*},\;a^{*}$, $b^{*}$ and $\Delta E_{ab}^{*}$) of injection-moulded samples of PLA and PBS with different 2, 4 and 6 wt% of natural pigment-based MBs developed.

Samples	$L^{*}$	$a^{*}$	$b^{*}$	$\Delta E_{ab}^{*}$
PLA/2%MB_Y	75.75	−9.57	65.32	---
PLA/4%MB_Y	76.26	−9.52	66.56	1.34
PLA/6%MB_Y	75.60	−6.18	67.38	3.97
PLA/2%MB_R	59.61	6.55	3.58	---
PLA/4%MB_R	60.46	7.29	4.11	1.25
PLA/6%MB_R	63.66	9.92	6.16	5.87
PLA/2%MB_G	29.31	−4.96	0.89	---
PLA/4%MB_G	29.96	−5.00	0.65	2.61
PLA/6%MB_G	29.82	−4.97	1.08	4.09
PBS/2%MB_Y	66.18	5.46	56.85	---
PBS/4%MB_Y	68.18	6.24	58.75	2.87
PBS/6%MB_Y	68.00	7.19	59.57	3.70
PBS/2%MB_R	79.11	9.64	6.61	---
PBS/4%MB_R	78.05	12.86	7.29	3.46
PBS/6%MB_R	77.52	13.86	7.32	4.57
PBS/2%MB_G	43.82	−9.32	2.83	---
PBS/4%MB_G	41.35	−9.04	2.03	3.88
PBS/6%MB_G	39.94	−8.65	1.72	5.05
PBS/2%MB_B	76.35	−3.65	−2.41	---
PBS/4%MB_B	74.38	−2.91	−5.67	3.88
PBS/6%MB_B	73.24	−3.37	−6.38	5.05

**Table 8:** Mechanical properties, Young’s modulus (E), tensile strength (σ_M_), elongation at tensile strength (Ɛ_M_), tensile strength at break (σ_R_), elongation at break (Ɛ_R_) and Charpy impact strength of PLA with the natural pigment-based MBs developed.

Samples	Young’s Modulus (MPa)	σ_M_ (MPa)	Ɛ_M_ (%)	σ_R_ (MPa)	Ɛ_R_ (%)	Charpy Impact Strength (kJ/m²)
As-received PLA	1710 ± 20.2	65.6 ± 0.3	3.8 ± 0.1	59.3 ± 2.4	4.5 ± 0.5	18.21 ± 1.5
PLA/2%MB_Y	1730 ± 18.1	65.8 ± 0.9	3.9 ± 0.1	60.9 ± 5.1	4.6 ± 0.6	19.02 ± 2.1
PLA/4%MB_Y	1690 ± 43.6	65.1 ± 0.5	3.9 ± 0.03	55.3 ± 1.8	5.2 ± 0.6	24.96 ± 2.7
PLA/6%MB_Y	1670 ± 19.9	64.0 ± 0.3	3.9 ± 0.03	59.9 ± 3.6	4.3 ± 0.5	26.12 ± 2.8
PLA/2%MB_R	1680 ± 41.2	66.7 ± 0.9	3.9 ± 0.04	63.0 ± 3.9	4.5 ± 0.5	14.98 ± 0.8
PLA/4%MB_R	1670 ± 58.5	64.8 ± 0.3	3.8 ± 0.1	61.1 ± 3.6	4.4 ± 0.7	14.38 ± 1.0
PLA/6%MB_R	1640 ± 23.3	63.7 ± 0.4	3.8 ± 0.02	58.2 ± 1.3	4.5 ± 0.3	14.15 ± 1.1
PLA/2%MB_G	1660 ± 36.4	62.1 ± 0.4	3.7 ± 0.04	53.3 ± 1.3	5.0 ± 0.3	17.79 ± 2.2
PLA/4%MB_G	1610 ± 29.4	65.3 ± 0.4	3.8 ± 0.4	54.2 ± 3.1	4.9 ± 1.0	17.76 ± 1.9
PLA/6%MB_G	1650 ± 48.0	64.9 ± 0.3	3.3 ± 0.3	53.8 ± 3.7	4.2 ± 0.3	19.02 ± 2.4

**Table 9:** Mechanical properties, Young’s modulus (E), tensile strength (σ_M_), elongation at tensile strength (Ɛ_M_), tensile strength at break (σ_R_), elongation at break (Ɛ_R_), and Charpy impact strength of PBS with the natural pigment-based MBs developed.

Samples	Young’s Modulus (MPa)	σ_M_ (MPa)	Ɛ_M_ (%)	σ_R_ (MPa)	Ɛ_R_ (%)	Charpy Impact Strength (kJ/m²)
As-received PBS	392 ± 17.2	34.5 ± 0.2	16 ± 0.7	26.5 ± 2.1	18 ± 0.5	5.17 ± 0.18
PBS/2%MB_Y	394 ± 24.3	34.7 ± 0.5	16 ± 1.60	27.4 ± 3.1	18 ± 0.6	5.07 ± 0.25
PBS/4%MB_Y	383 ± 27.2	34.4 ± 0.2	17 ± 0.20	25.7 ± 0.7	19 ± 0.5	4.98 ± 0.35
PBS/6%MB_Y	374 ± 12.3	34.1 ± 0.4	17 ± 0.33	29.9 ± 3.1	18 ± 0.6	5.13 ± 0.44
PBS/2%MB_R	383 ± 30.5	35.0 ± 0.2	17 ± 0.20	33.2 ± 0.4	19 ± 0.5	4.94 ± 0.42
PBS/4%MB_R	384 ± 43.9	34.2 ± 0.2	16 ± 0.38	31.7 ± 0.7	18 ± 0.7	4.96 ± 0.76
PBS/6%MB_R	397 ± 28.0	34.5 ± 0.2	16 ± 0.14	32.2 ± 0.4	16 ± 0.1	4.56 ± 0.23
PBS/2%MB_B	392 ± 25.7	34.0 ± 2.0	14 ± 3.50	32.7 ± 1.2	19 ± 0.9	4.86 ± 0.32
PBS/4%MB_B	406 ± 67.6	35.2 ± 0.1	16 ± 0.25	32.8 ± 0.58	19 ± 0.4	5.02 ± 0.42
PBS/6%MB_B	391 ± 46.9	34.7 ± 0.1	16 ± 0.34	32.5 ± 0.393	19 ± 1.1	5.29 ± 0.36
PBS/2%MB_G	436 ± 11.7	36.4 ± 0.3	16 ± 0.19	33.8 ± 0.733	19 ± 0.6	5.54 ± 0.51
PBS/4%MB_G	417 ± 13.2	35.8 ± 0.2	17 ± 0.14	33.6 ± 0.7	19 ± 0.5	5.20 ± 0.26
PBS/6%MB_G	391 ± 18.8	34.7 ± 0.4	16 ± 0.33	30.6 ± 3.4	18 ± 0.8	4.75 ± 0.31

## Data Availability

The original contributions presented in the study are included in the article, further inquiries can be directed to the corresponding author/s.
